# Frozen Autoclaved Sorghum Enhanced Colonic Fermentation and Lower Visceral Fat Accumulation in Rats

**DOI:** 10.3390/nu12082412

**Published:** 2020-08-12

**Authors:** Samanthi W. Pelpolage, Atsushi Yoshida, Ryuji Nagata, Kenichiro Shimada, Naoki Fukuma, Hiroki Bochimoto, Tetsuo Hamamoto, Michiyo Hoshizawa, Koichi Nakano, Kyu-Ho Han, Michihiro Fukushima

**Affiliations:** 1 Department of Life and Food Sciences, Obihiro University of Agriculture and Veterinary Medicine, West 2-11, Inada, Obihiro 080–8555, Hokkaido, Japan; samanthipelpola@gmail.com (S.W.P.); yield0000@gmail.com (A.Y.); kshimada@obihiro.ac.jp (K.S.); boc.10rj24@gmail.com (R.N.); n.fukumax@obihiro.ac.jp (N.F.); kyuho@obihiro.ac.jp (K.-H.H.); 2 Research Center for Global Agromedicine, Obihiro University of Agriculture and Veterinary Medicine, West 2-11, Inada, Obihiro 080-8555, Hokkaido, Japan; 3 Division of Aerospace Medicine, Department of Cell Physiology, The Jikei University School of Medicine, 3-25-8 Nishishimbashi, Minatoku, Tokyo 105-8461, Japan; botimoto@jikei.ac.jp; 4 U.S. Grains Council, 11th Floor, Toranomon Denki Building No. 3, 1-2-20 Toranomon, Minato-ku, Tokyo 105-0001, Japan; thamamoto@grains.org (T.H.); mhoshizawa@grains.org (M.H.); 5 Nakano Industry Co., Asahishinmachi 33-25 Takamatsu, Kagawa 760-0064, Japan; nakano@nakano-sangyo.co.jp

**Keywords:** colonic fermentation, lipid metabolism, sorghum, visceral fat

## Abstract

As raw sorghum is not able to influence considerable colonic fermentation despite its higher resistant starch (RS) content, our study aimed to investigate the effects of frozen autoclaved sorghum on colonic fermentation. Fischer 344 rats were fed frozen cooked refined (S-Rf) and whole (S-Wh) sorghum diets and were compared against α-corn starch (CON) and high amylose starch (HAS) fed rats for zoometric parameters, cecal biochemical and microbiological parameters. Sorghum fed rats exhibited significantly lower feed intake and visceral adipose tissue mass compared to CON. Bacterial alpha diversity was significantly higher in the sorghum fed rats compared to HAS and the two sorghum fed groups clustered together, separately from HAS and CON in the beta diversity plot. Serum non-High Density Lipoprotein cholesterol and total cholesterol in S-Rf group were significantly lower compared to CON, while total fecal bile excretion was also significantly higher in the two sorghum fed groups. Lower visceral adiposity was correlated with lower feed intake, RS content ingested and cecal short chain fatty acid (SCFA) contents. Thus, higher RS inflow to the colon via frozen autoclaved sorghum might have influenced colonic fermentation of RS and the resultant SCFA might have influenced lower adiposity as manifested by the lower body weight gain.

## 1. Introduction

Decreased incidence of non-communicable diseases such as obesity and related co-morbidities has been linked with the beneficial effects of gut microbial fermentation of dietary resistant polysaccharides and fermentation metabolites [1]. Short chain fatty acids (SCFA) are mostly appreciated as the key mediators of beneficial physiological effects via binding with respective receptors and enhancing homo-neuronal or transcription factor mediated metabolic pathways [1]. Acetate is known to lower the accumulation of abdominal fat and protect from lipid accumulation in the liver, thus reduction in lipid content in adipose tissue and liver suggests an improved glucose tolerance [2]. Further, adenosine monophosphate kinase (AMPK) activation in adipose tissue by SCFA is known to decrease lipogenic flux, triglyceride catalysis, and increase fatty acid oxidation and thus reduce the free fatty acid release into and, its availability in, plasma [2]. Moreover, *n*-butyrate and propionate are found to activate intestinal gluconeogenesis, which in turn maintains energy homeostasis via regulation of food intake and enhancement of insulin sensitivity [1,3].

Resistant starch (RS) has a negligible contribution in terms of caloric supply, but it mediates more beneficial biochemical reactions upon reaching the colon [4]. RS that reaches the colon is known to be disintegrated by the colonic microbiota via their versatile enzyme repertoire, which exceeds the host enzyme capacity [4]. Being an anaerobic fermentation process, it yields SCFA as the main by-products that are known as signaling molecules, which mediate many biochemical reactions in the adipose tissue, liver, brain axis and the intestine itself [5]. The SCFA mediated upregulated or downregulated biochemical processes are known to exert beneficial health effects on the host, such as prevention of colon cancers, homeostasis of lipid and glucose metabolism, and prevention of fat accumulation, overweight and obesity, etc. [6,7].

RS content in processed foods depends mainly on the processing temperature and moisture availability (processing method) other than genetic factors such as native crystallinity [8]. Most of the commonly used thermal processing methods, such as steam cooking, autoclaving, boiling and baking, are known to increase the RS content [8,9,10]. Previous studies have reported significant increments in RS and total dietary fiber contents in wheat flour and native wheat starch by autoclaving, where the increment of dietary fiber in autoclaved wheat flour was attributed to the increment of RS [8]. RS3, the main type of RS found in processed starchy foods, known as retrograded starch, is known to be highly resistant to thermal and enzymatic degradation due to the highly ordered re-crystallized amylose [11]. The re-crystallized linear amylose chains, which form tightly packed double helices stabilized by inter chain H-bonds (junction bonds), hinder the accessibility of enzymes to starch molecules [1,11].

Sorghum (*Sorghum bicolor* L. Moench) is the fifth most cultivated cereal in the world and is known for its higher native RS content (12–21%) compared to other cereals [12,13]. Raw sorghum exhibited moderate colonic fermentation potential under in vitro and in vivo conditions as previously reported by the authors [14,15]. Cooking of sorghum has shown to increase the RS content due to decreased protein digestibility and enhanced strength of the amylose-lipid complex according to previous studies [13,16,17]. Thus, reduced ileal digestibility upon cooking of sorghum suggests a higher flow of RS into the colon, which might suggest an improved colonic fermentation of RS by microbiota. A recent study has shown that feeding of RS from autoclaved and retrograded sorghum was able to prevent body weight gain and improve blood lipids and beneficial gut microbiota in obese and overweight rats [18].

Sorghum being a very promising source of native RS reported to improve inherent RS content upon cooking, we hypothesized that autoclaving and freezing might increase RS content in sorghum, which might increase the starch flow into the colon and thus improve colonic fermentation. Cooking of grains being a common practice across most of cuisines, the ability to increase RS in food sources by cooking would be a noteworthy, inexpensive method that can be employed even in households as a strategy for mitigating non-communicable diseases, such as overweight, obesity and diabetes. Thus, we aimed to investigate the effect of frozen cooked sorghum on colonic fermentation characteristics and subsequent physiological effects in rats.

## 2. Materials and Methods

### 2.1. Preparation of Experimental Diets

Whole grain sorghum and refined grain sorghum were provided by the Nakano Industry Co. (Takamastu, Japan) and U.S. Grains Council (Tokyo, Japan). Sorghum grains were subjected to cooking (grains: water = 1:2) by autoclaving (121 °C; 20 min; SX-300 high pressure steam sterilizer, Tomy Seiko Co., Ltd., Tokyo, Japan). Cooked grains were stored overnight at −30 °C before they were freeze-dried (−80 °C, Eyela FDU-2100, Tokyo Rikkai Co., Ltd., Tokyo, Japan). Freeze-dried cooked grains were milled (mill aperture size; 1 mm, MRK-RETSCH, Cross Beater Mill, Giesbeek, The Netherlands) and stored at −30 °C until further analyses. The moisture content (Association of Official Analytical Chemists (AOAC) method 930.15), protein (AOAC 979.09) with a conversion factor of 6.25, lipid (AOAC 920.85) and ash (AOAC 923.03) were analyzed for the two freeze-dried samples. Resistant starch contents were determined by Megazyme resistant starch assay procedure (K-RSTAR 08/11, Wicklow, Ireland) according to AOAC method 2002.02. Amylose, amylopectin and total starch contents were determined by Megazyme amylose/amylopectin assay (K-AMYL 07/11) according to the method by Yun and Matheson [19]. Dietary fiber in the two samples was measured according to AOAC 2011.25 method. Proximate composition of frozen cooked sorghum is provided in Table S1. All chemicals used were of analytical grade. Four experimental diets, α-corn starch (CON), high amylose starch (HAS; 30% *w/w* HS-7, J-Oil Mills Co., Ltd., Tokyo, Japan), frozen cooked whole sorghum (S-Wh; 30% *w/w*), and frozen cooked refined sorghum (S-Rf; 30% *w/w*) were prepared according to the AIN-93G diet guidelines by Oriental Yeast Co., Ltd., (Tokyo, Japan) (Table S2).

### 2.2. Animal Experimental Design, Care for Laboratory Animals and Post-Mortem Excision of Organs

The animal experiment was conducted according to the guidelines of “Guide for the Care and Use of Laboratory Animals” and all the procedures were approved by the Animal Care and Experiment Committee of Obihiro University of Agriculture and Veterinary Medicine (License No.: 18–86). The rat strain (Fischer 344) was chosen as the experimental animal model in this study, as it has been previously used in nutrition and dietary research as a suitable model for studying body weight gain, adipose fat accumulation and effects of dietary fiber [20,21,22,23].

Twenty four Fischer 344 male rats (seven weeks old; average body weight 135–165 g) were purchased from Charles River Laboratories Japan Inc., (Yokohama, Japan). After acclimatization for one week on a commercial diet (Standard powder diet for mouse, rat, hamster, Oriental Yeast Co.) rats were grouped into four similar body weight groups (≃187 g). Following grouping, rats were cared and maintained as previously mentioned in Pelpolage et al. [24] with free access to experimental diets (≃25 g) and ad libitum water (≃150 mL). The cages were maintained at 23 ± 1 °C temperature and 60 ± 5% relative humidity under a 12 h light/dark cycle and each rat was individually housed.

Weekly, body weight gain and daily feed intake were measured. Following a 12 h fasting period blood (≈1 mL) was collected weekly and the separated serum via centrifugation (8000 rpm; 15 min; two times, CFA-12, Iwaki Glass Co., Ltd., Tokyo, Japan) was stored at −80 °C until biochemical analysis. Feces were collected during the last four days of the experimental period for fecal lipid analysis. Fresh feces were collected directly from anus for fecal moisture analysis. After the experimental period of four weeks, the final body weight was measured at 08.00 h and the animals were sacrificed inside their home cages, by an intraperitoneal injection of narcotic (sodium pentobarbital, 40 mg/kg body weight, Abbott Laboratories, Chicago, IL, USA) assuring minimal level of suffering.

Following sacrifice, cecum, liver, epididymal and perirenal adipose tissue masses were excised and weighed. After measuring total cecal weight, cecal content weight and cecal tissue weight, a part of the cecal content (≃1 g) was diluted (×5) in sterilized distilled water for pH measurement [25] and other analyses, while the rest was stored at −80 °C. Liver was frozen in liquid nitrogen and stored at −30 °C for liver lipid analysis. Mesenteric adipose tissue was fixed in freshly prepared 10% neutral buffered formalin and was stored at 4 °C until staining. 

### 2.3. DNA Extraction, Next-Generation Sequencing (NGS) and the Analysis of 16S Ribosomal RNA (16S rRNA) Gene Sequences in Cecal Content

Extraction of bacterial DNA from the cecal content samples (stored at −80 °C) by modified phenol-free repeated bead beating plus column (RBB + C) method, purification of extracted genomic DNA (QIAamp DNA Stool Mini Kit, QIAGEN, Valencia, CA, USA) and measurement of concentration of extracted community DNA (Nano Drop 2000c spectrophotometer, Thermo Fisher Scientific, Tokyo, Japan) were conducted as previously mentioned [24]. Finally, the extracted and purified genomic DNA concentration was adjusted to 5 ng/µL with Tris-EDTA buffer. 

The preparation of 16S rRNA gene amplicon required for sequencing was carried out following the method described in the 16S Metagenomics Sequencing Library Preparation Guide (Part # 15044223 Rev. B). During the first stage polymerase chain reaction (PCR), V3 and V4 variable regions of 16S rRNA gene were amplified using the following bacterial overhang adapters and universal primers; forward primer and the overhang adapter (5′-TCG TCG GCA GCG TCA GAT GTG TAT AAG AGA CAG CCT ACG GGN GGC WGC AG-3′) and reverse primer and the overhang adapter (5′-GTC TCG TGG GCT CGG AGA TGT GTA TAA GAG ACA GGA TTA CHV GGG TAT CTA ATC C-3′). Illumina sequencing adapters and dual index barcodes were multiplexed to the first stage PCR amplicons using Nextera^®^ XT Index Kit (Illumina Inc., San Diego, CA, USA) during the second stage PCR. The successful PCR products were pooled in one tube with equal volumes and were subjected to paired-end sequencing by Illumina MiSeq System (Illumina Inc.), after measuring the concentration of the second stage PCR products (Quantus™ fluorometer, QuantiFluor^®^ dsDNA System, Promega, Madison, WI, USA).

The analysis of retrieved raw 16S rRNA gene sequences was conducted according to the method reported in Warren et al. [26]. The biome table was normalized using an equal subsampling size of 13,674 sequences. Preparation of Principle Coordinate Analysis (PCoA) plot in Quantitative Insights Into Microbial Ecology (QIIME2, version 1.9.1) and hierarchical clustering plots at phylum, genus and species levels (Calypso version 8.72) were also similar to the methods described in Pelpolage et al. [24].

### 2.4. Rat Cecal SCFA Analysis by High Performance Liquid Chromatography (HPLC)

SCFA content in the cecal digesta of rats were analyzed by HPLC (LC-10AD, Shimadzu Co., Kyoto, Japan). Samples for HPLC were prepared according to the method described in Han et al. [25]. Briefly, 450 µL of cecal suspension (mentioned in Section 2.2) supernatant (centrifuged; 3000 rpm, 4 °C, 10 min) was conjugated with 1 mL of 0.5 N HCLO_4_ (60% *v/v*). Upon centrifugation under the same conditions, 300 µL of the supernatant was filtered through a micron membrane filter (0.45 μm; DISMIC-03CP, Advantec, Toyo Roshi Kaisha, Tokyo, Japan) and was used in HPLC. Analytical specifications were as follows; column, RSpak KC-811 (8.0 mm × 300 mm, Shodex, Tokyo, Japan); eluent and flow rate, 2 mM HClO_4_ at 1 mL/min; column temperature, 47 °C; reaction reagent and flow rate, ST3-R (×10 diluted, Cat. No. F56120000, Shodex) at 0.5 mL/min; UV detector wavelength, 450 nm. Quantification of SCFA concentration was performed according to the method reported in Pelpolage et al. [24].

### 2.5. Fecal and Liver Lipid Analysis

Total lipid fractions in feces (frozen; −80 °C) and liver were extracted with chloroform-methanol (2:1 *v/v*) and chloroform-methanol-water (1:1:0.9 *v/v*) solvents, respectively, as previously mentioned [27]. In brief, the total lipid content was measured gravimetrically upon completely drying the organic solvents obtained from the three sequential extractions with either chloroform/methanol (feces) or chloroform/methanol/water (liver) using the Büchi rotavapor (R-114, Büchi, Tokyo, Japan). Liver triglyceride and cholesterol fractions were measured using commercially available kits (Wako Pure Chemical Industry, Ltd., Osaka, Japan). Fecal neutral sterol and acidic sterol fractions were quantified by gas liquid chromatography (GC-2014, Shimadzu Co., Tokyo, Japan) as previously mentioned [28,29]. The fecal total lipid fraction separated in the previous step was first subjected to methylation with methanol: water (10:9) solvent. Methylated sample was dissolved in 0.1 mL of chloroform: methanol (2:1) solvent to be used in thin-layer chromatography (TLC) and the sample separated from TLC was acetylated (pyridine: anhydrous acetic acid = 1:1). Acetylated sample was purified (methanol: water = 10:9), centrifuged (2000 rpm, 10 min) and dried completely (Büchi rotavapor) before dissolving in 100 µL acetone to be used in gas chromatography. Analytical specifications were as follows: column, BD17 capillary column (0.25 mm × 30 m; J & W Scientific Folsom, CA, USA); carrier gas, N_2_ (1.41 mL/min, 1.38 mL/min; neutral sterol, acidic sterol, respectively); inlet pressure, 187, 187.5 kPa (neutral and acidic sterol, respectively): temperature of column, injection port, detector, 260, 270 °C (neutral and acidic sterol, respectively), 300 °C, 300 °C, respectively; detector, flame ionization detector (FID-2014, Shimadzu Co., Tokyo, Japan).

### 2.6. Mesenteric Adipocyte Staining

Adipocyte area (μm^2^) is presented as the average area of the adipocytes in three randomly taken images (magnification 10×, BA210E, Shimadzu Co., Tokyo, Japan) per each 4 µm thick paraffin-embedded tissue sections stained with hematoxylin and eosin as previously mentioned [30]. In a nutshell, adipose tissue samples preserved in 10% buffered formalin were dehydrated in a series of ethanol solutions (70% to 100%) and fixed in xylene for 30 min on a shaker. Later the paraffin blocks of xylene-fixed tissue samples were prepared and a 4 µm thick tissue cross-section was obtained (microtome replacement blade S35, KN3321485) and was fixed onto a labelled glass slide. Glass slides with fixed tissue cross-sections were immersed in a gradient series of xylene (xylene I to III, AbA1 I to II) and later in a gradient series of ethanol solutions (100% to 70%) and finally in distilled water, for 1 min in each. Later the tissue samples were stained in hematoxylin and then in 1% eosin. Followed by staining in hematoxylin and eosin, the tissue samples were further fixed in a gradient series of ethanol (70% to 100%) and later in a gradient series of xylene in divergent order. After staining, the tissue sections on the glass slides were covered with a cover slip and allowed to dry overnight. Images were processed using Fuji Image J software (ImageJ bundled with 64-bit Java 1.8.0_112). 

### 2.7. Serum Biochemical Analysis

Serum biochemical profile was analyzed using Toshiba TBA-120FR auto analyzer (Toshiba Medical Systems Co., Tochigi, Japan).

### 2.8. Statistical Analysis

All zoometric and biochemical data (for six rats per group) were analyzed for their significance (*p* ˂ 0.05) by analysis of variance (ANOVA) paired with Tukey’s test using SPSS statistical software version 17.0 (IBM Co., Armonk, NY, USA). Pearson’s correlation analysis was conducted using SPSS to identify correlations among the parameters. Statistical significance of alpha diversity indices was determined by ANOVA coupled with Tukey’s test (SPSS). Relative abundance and statistical significance of phyla, genera and species among the four groups were compared using Kruskal-Wallis H test in Calypso (version 8.72). A *p* value less than 0.05 was considered as statistically significant.

## 3. Results

### 3.1. Zoometric Parameters, Feed Intake and Organ Weights

**Figure 1:**
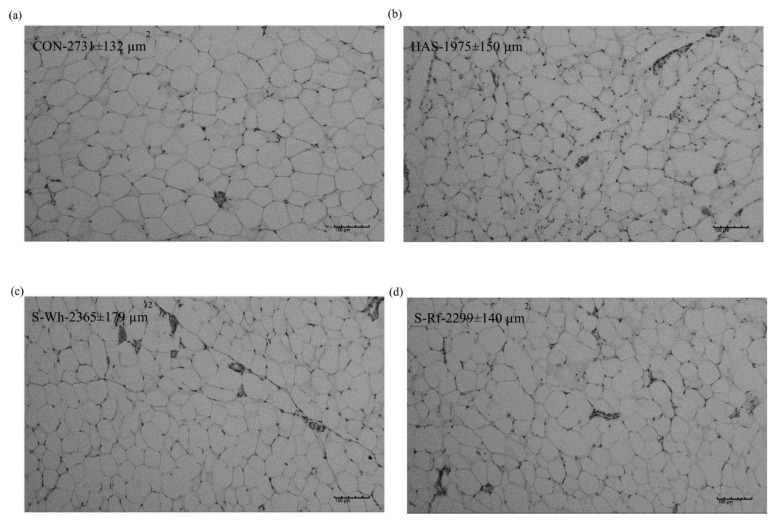
Mesenteric adipocyte area of rats fed (**a**) CON, α-corn starch (**b**) HAS, high amylose starch (**c**) S-Wh, frozen cooked whole white sorghum and (**d**) S-Rf, frozen cooked refined white sorghum. Values presented are mean ± SE (*n* = 6). Statistical significance was determined by ANOVA paired with Tukey’s test (*p* < 0.05).

Zoometric and organ weight data are presented in Table 1. At the end of the experimental period, final body weight was significantly (*p* < 0.05) lower in the HAS group compared to CON group, while both sorghum groups gained a comparatively lower final body weight compared to CON (S-Wh, S-Rf; *p* = 0.27, 0.19). Body weight gain also followed the same trend as the final body weight. Feed intake was significantly lower in HAS, S-Wh and S-Rf compared to the CON group. HAS group reported the lowest perirenal and epididymal adipose tissue weights (per 100 g body weight) and total visceral adipose tissue weight (per 100 g body weight) among the groups. The two sorghum fed groups had significantly lower epididymal, perirenal and total visceral adipose tissue weights (per 100 g body weight) compared to CON group. Adipocyte size was also comparatively (S-Wh, S-Rf; *p* = 0.132, 0.049) lower in the two sorghum groups compared to CON group (Figure 1). These results suggested that lower feed intake observed in the sorghum fed groups might have been the reason for lower visceral adiposity and body weight gain, thus sorghum diets had influenced a lower appetite in rats. Cecal parameters (cecal weight, cecal content weight and cecal tissue weight) were significantly higher in the HAS group, while the other three groups reported significantly lower values, yet similar to each other (Table 1), suggesting a prominent cecal fermentation in the HAS group.

### 3.2. Microbial Community DNA Data

#### 3.2.1. Alpha and Beta Diversities

**Figure 2:**
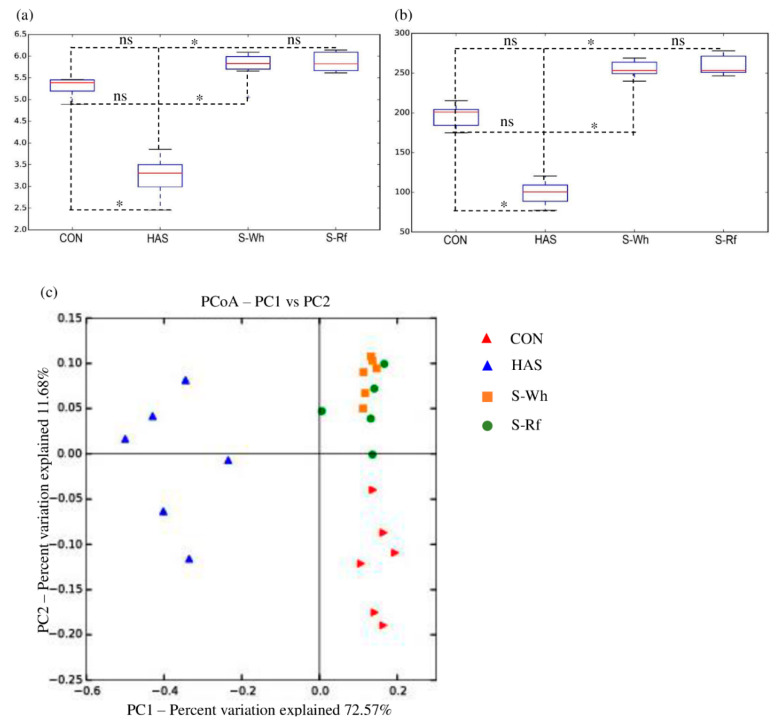
Box and whisker plot of (**a**) Shannon’s diversity index and (**b**) Observed species index (**c**) Weighted UniFrac Principle Coordinate Analysis (PCoA) plot for the β-diversity. For (**a**) and (**b**), values presented are mean ± SE (*n* = 6) and statistical significance was determined by ANOVA paired with Tukey’s test (*p* < 0.05). β-diversity was determined by the weighted UniFrac distance metric in QIIME. (CON, α-corn starch; HAS, high amylose starch; S-Wh, frozen cooked whole white sorghum; S-Rf, frozen cooked refined white sorghum; ns, not significant).

Metagenomic analysis of microbial DNA revealed significantly (*p* < 0.05) higher alpha diversity (Shannon index and observed species index) in the two sorghum samples compared to the positive control HAS (Figure 2a,b), which suggested the presence of a more diverse microbial community and an evenly distributed abundance of the observed species (evenness). Thus, a higher alpha diversity in the two sorghum groups might suggest the positive impact of the frozen cooked sorghum diets on maintaining a diverse microbiota, possibly due to the availability of suitable substrates to feed a wide variety of microbiota. A higher diversity also reflected the presence of a healthy microbiota in the two sorghum groups. PCoA plot (beta diversity) exhibited evidence for distinct microbial compositions among the diet groups by clear cluster formation (Figure 2c). The two frozen cooked sorghum groups clustered together, while CON and HAS groups clustered separately from sorghum groups and each other. PCoA plot suggested that the different diets had significant effects in shaping the microbial composition, yet the whole or refined nature of sorghum seemed not to have had any significant influence on determining the microbial composition in the sorghum fed groups.

#### 3.2.2. Taxonomic Diversity

**Figure 3:**
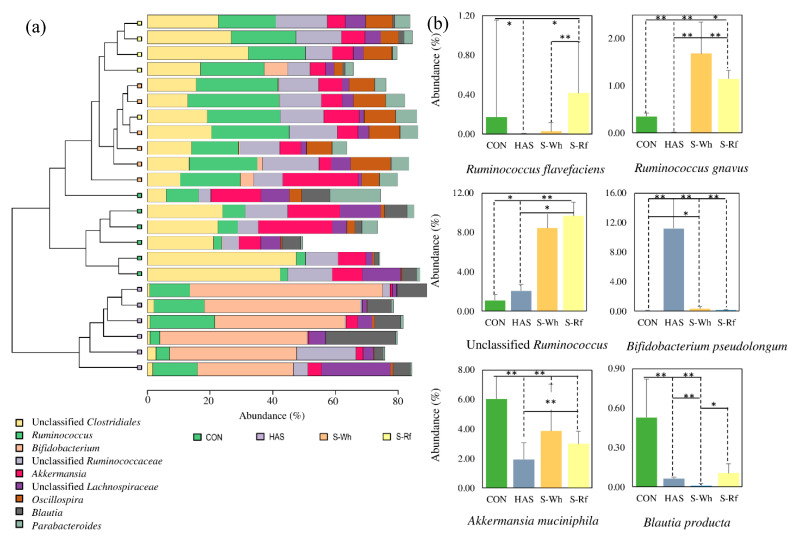
(**a**) Clustered bar chart at genus level for cecal microbiota. (**b**) Rank test bar charts for the relative abundance (median ± SE) of selected microbial species in the rat cecal digesta. For (**b**), Statistical significance was determined by Kruskal-Wallis H test in Calypso (version 8.72) (* *p* < 0.05; ** *p* < 0.01). (Abbreviations: CON, α-corn starch; HAS, high amylose starch; S-Wh, frozen cooked whole white sorghum; S-Rf, frozen cooked refined white sorghum).

Clear cluster formation depending on the diet factor was further revealed by the clustered bar chart obtained at genus level (Figure 3a), where the groups were similarly clustered as seen in Figure 2c. As presented in Figure 3a, the two sorghum groups could be characterized by the higher abundance of genus *Ruminococcus*, while HAS group was characterized by genus *Bifidobacterium*.

The characteristic microbial genera in sorghum and HAS might indicate the potential major substrates available for microbial utilization in each group, which might have been the main reason for influencing the conventional dietary fiber fermenters and RS fermenters in sorghum groups and HAS group. At species level *R. flavefaciens*, *R. gnavus*, unclassified *Ruminococcus* abundances were significantly higher in the two sorghum groups (Figure 3b). Further, in the HAS group, *B. pseudolongum*, unclassified *Blautia* and, in the CON group, *Akkermansia muciniphila*, *Blautia producta*, *Lactococcus gravieae* and *Coprococcus eucactus* were found in higher abundance.

Albeit that the total dietary fiber content in all diet groups was similar (50 g/kg diet), a higher (*p* < 0.05) content had been ingested by the CON fed animals (19.2 g/total feed intake) in the form of cellulose (non-fermentable fiber). On the other hand, sorghum fed animals had ingested dietary fiber from sorghum 4.0 and 1.5 g/total feed intake, via S-Wh and S-Rf diets, respectively, in addition to cellulose (S-Wh, S-Rf; 13.3, 15.5 g/total feed intake). Further, sorghum fed animals had ingested RS contents of 3.8 and 4.8 g/total feed intake via S-Wh and S-Rf, respectively, in comparison to 0 and 37.7 g/total feed intake via CON and HAS, respectively. Thus, the cluster formation, differently abundant genera and significantly higher abundance of specific microbial species in each group proved the fact that the above mentioned compositional differences in the test diets might have been significant driving factors on the microbial composition in cecum.

### 3.3. Cecal Short Chain Fatty Acid Content and pH

Acetate, butyrate and total SCFA contents (per cecum) were significantly (*p* < 0.05) higher in the HAS group compared to the other three groups, while propionate content was similar across the groups (Table 2). In comparison to S-Wh group, S-Rf group exhibited comparatively higher individual and total SCFA contents (acetate, propionate, butyrate and total SCFA; *p* = 0.11, 0.22, 0.07 and 0.11, respectively). Cecal content pH was significantly lower in the HAS group compared to other groups, while S-Rf had comparatively lower cecal pH compared to S-Wh (*p* = 0.72). Cecal pH was negatively correlated with the total SCFA content (*r* = −0.78; *p* < 0.01) and also with the individual SCFA contents (Table S3), which indicated the effect of SCFA on acidifying the cecal environment. Thus, SCFA production might have caused the reduction of cecal pH and at the same time cecal hypertrophy as suggested by the significant correlations between cecal parameters and SCFA concentrations (Table S3). Comparatively higher acetate (*p* = 0.12), *n*-butyrate (*p* = 0.07) and total SCFA (*p* = 0.38) contents in the HAS group can be attributed to the very high RS content (ten-fold higher) compared to the S-Rf group.

### 3.4. Liver Lipid Profile, Fecal Moisture and Dry Matter Contents, Lipid and Bile Acid Profiles

#### 3.4.1. Liver Lipid Profile

Liver weight, liver total lipids and triglyceride contents were similar among the four groups, yet the liver cholesterol content was significantly (*p* < 0.05) lower in the HAS group, while CON and S-Wh groups had comparatively (*p* = 0.65) and significantly (*p* = 0.04) lower content compared to S-Rf group, respectively (Table 3). Higher cholesterol content in the S-Rf group suggested either a higher clearance of extrahepatic cholesterol via reverse cholesterol transport by HDL-cholesterol or higher uptake of very low density lipoprotein (VLDL) remnants and LDL-cholesterol by the liver.

#### 3.4.2. Fecal Moisture, Dry Matter, Lipid and Bile Acid Profiles

Fecal dry matter content was significantly (*p* < 0.05) higher in the HAS group, while it was significantly lower in the two sorghum groups, but there was no significant difference in the fecal moisture content (Table 3). Higher RS content along with the cellulose fraction in the HAS group could be responsible for the higher fecal dry mass content, as fecal mass positively correlates with dietary fiber intake. However, in this study the fecal mass did not correlate with either total dietary fiber or cellulose content. Further, RS is also well-known to increase fecal wet and dry weight by a factor of 1.8, largely due to the increment of bacterial biomass, where this study also revealed a positive correlation (*r* = 0.51, *p* < 0.05). Significantly lower fecal dry weight in the two sorghum groups might suggest the excretion of a lower quantity of non-fermentable substrates, thus certain components in sorghum dietary fiber might have been catabolized by the colonic microbiota.

On the other hand, fecal lipid profile was similar among the four diet groups, despite the higher fecal dry weight in the HAS group. However, there was clearly a higher total lipid content excreted in the HAS group followed by S-Wh group compared to CON (*p* = 0.05, 0.80, respectively) and S-Rf (*p* = 0.05, 0.38, respectively) groups. Further, comparatively higher cholesterol contents were also found excreted via feces in S-Rf group followed by HAS group compared to CON (*p* = 0.27, 0.11, respectively) and S-Wh (*p* = 0.26, 0.02, respectively) groups. Generally total fecal lipid content, which is around 8.7 to 16% dry weight basis, positively correlates with fiber intake, as similarly seen in the present study (*r* = 0.52, *p* < 0.01).

Fecal total bile excretion was significantly (*p* < 0.05) higher in S-Wh group, while it was comparatively higher in the HAS (*p* = 0.08) and S-Rf (*p* = 0.60) groups compared to CON (Table 3). There were no significant differences in the individual (cholic acid and chenodeoxycholic acid) and total primary bile acid contents, yet they were comparatively higher in the HAS (*p* = 0.07, 0.13, respectively) and S-Rf (*p* = 0.23, 0.34, respectively) groups compared to CON. On the other hand, individual and total secondary bile acid contents were significantly different among the groups (Table 3). Deoxycholic acid content was significantly higher in the S-Wh group, while it was comparatively higher in the S-Rf group compared to CON (*p* = 0.23) and HAS (*p* = 0.03). Lithocholic acid content was significantly higher in the S-Rf group, while it was comparatively higher in the S-Wh group compared to CON (*p* = 0.09) and HAS (*p* < 0.01). Total secondary bile acid content was significantly higher in the two sorghum fed groups compared to CON and HAS. Further, the higher fecal secondary bile acid contents in the two sorghum groups indicated a higher bacterial conversion of primary bile acids, which reflected that there might have been more primary bile acids being passed down to the colon, binding with dietary fiber that might have been liberated when dietary fiber was catabolized by bacteria.

### 3.5. Serum Lipid Profile

**Figure 4:**
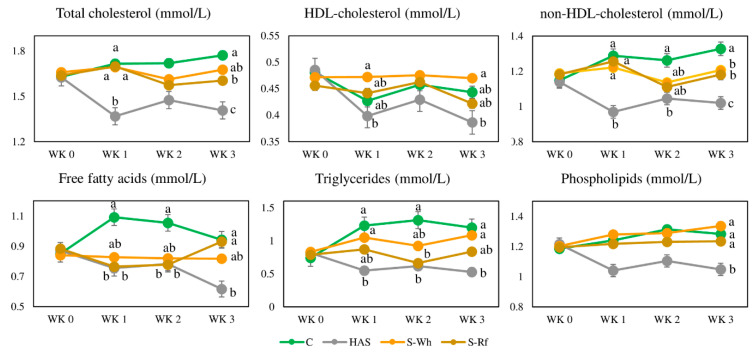
Temporal variation in the serum lipid profile of rats. Values presented are mean ± SE (*n* = 6). Different lowercase letters indicate significant (*p* < 0.05) differences among the groups. (Abbreviations: CON, α-corn starch; HAS, high amylose starch; S-Wh, frozen cooked whole white sorghum; S-Rf, frozen cooked refined white sorghum).

Serum lipid parameters of the four groups are presented in Figure 4. HAS group exhibited significantly lower serum lipid parameters compared to the other three groups at the end of the experimental period. Serum total cholesterol in S-Rf and non-HDL-cholesterol (non-HDL-Cholesterol = total cholesterol—HDL-cholesterol) in both groups were significantly (*p* < 0.05) lower compared to the CON group at wk. 3.

HDL-cholesterol and free fatty acids contents in the S-Wh group remained constant throughout the experimental period, while free fatty acids content was significantly lower between wk. 0 to 2 in the S-Rf group, which drastically increased during the next week. Serum triglyceride content varied similarly in the two sorghum groups, which were similar to that of the CON at the end. Serum lipid profile also exhibited a clear cross-section of the lipid homeostasis among adipose tissue, liver and extrahepatic tissue, where lipid homeostasis seemed improved in the sorghum groups compared to CON group.

## 4. Discussion

Among many extrinsic and intrinsic factors that influence body weight, diet is one of several factors that an individual has a complete control over, thus diet and body weight management has become a lucrative commercial avenue with the escalating increment of obesity and related co-morbidities globally. Albeit that the etiology of obesity is multifactorial, the most common obesity phenotype is the excessive fat accumulation in the adipose tissue depots, skeletal muscles and liver as a result of a long-term positive energy balance that can be implicated by body weight gain [2]. A positive energy balance might result due to both increased energy consumption and/or decreased energy expenditure, which might be influenced by genetic factors, age/development milestones, ethnicity and physical activity level, etc. [31]. Even though considerable controversy remains over whether the dietary composition or total energy intake exert a larger influence on body weight gain, a high fat diet is well-known to influence adiposity in both animals and humans [2]. On the other hand, diets rich in complex carbohydrates that fall under the prebiotic and/or soluble dietary fiber group have been in the spotlight for their anti-obesity effect in relation to diluted calorie intake and beneficial outcomes on energy metabolism in the body by the colonic breakdown of complex carbohydrates by gut colonizing bacteria [4].

**Figure 5:**
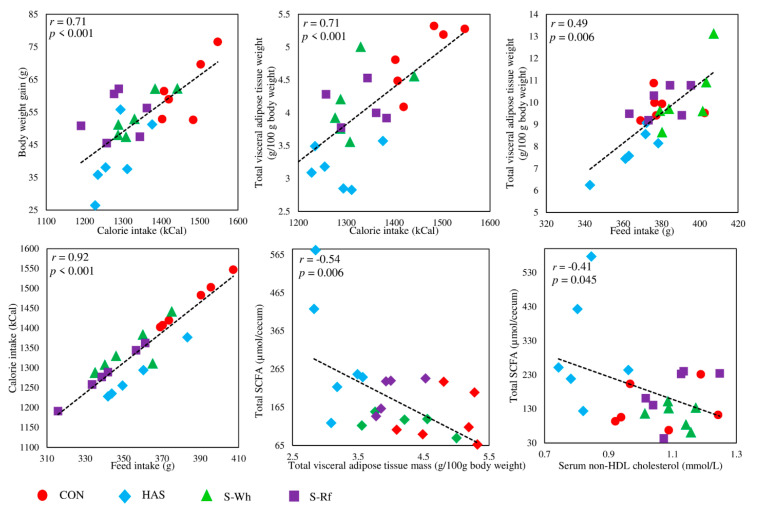
Scatter plots for selected important Pearson’s correlations (Abbreviations: CON, α-corn starch; HAS, high amylose starch; S-Wh, frozen cooked whole white sorghum; S-Rf, frozen cooked refined white sorghum).

In this study, the two sorghum fed groups reported comparatively lower body weights/gains compared to the negative control group (CON), and most significantly (*p* < 0.05) lower visceral fat mass and serum non-HDL cholesterol level. Lower calorie intake (calorie content/100 g diet (kCal): CON, 380; S-Wh, 384; S-Rf, 377) due to lower (*p* < 0.05) feed consumption might have been a reason behind the comparatively lower body weight gain in the S-Wh (*p* = 0.43) and S-Rf (*p* = 0.52) groups compared to CON [32], which was further strengthened by the positive correlations observed for calorie intake with body weight gain (Figure 5) and feed intake (Figure 5). Further, lower calorie intake could also have been a reason behind the lower (*p* < 0.05) visceral fat accumulation in rats as suggested by the positive correlation between calorie intake and visceral adipose tissue mass (Figure 5), which might have resulted in a lower quantity of excess energy or positive energy balance that demands storage within the adipose tissue. Positive correlation between the calorie intake and the adipocyte size (*r* = 0.45; *p* = 0.029) also might further strengthen the above fact. Thus, it was clear that the lower calorie intake due to the reduced appetite might have been a major reason behind the lower total visceral adiposity and lower body weight gain in sorghum fed rats. In any case, a clear reason for the lower feed intake in the sorghum groups could not be identified, except for a negative correlation with the sorghum dietary fiber fraction (*r* = −0.41; *p* = 0.04). Albeit that RS intake was not directly correlated with the feed intake, it was clear that RS intake via diet had been a major player in reducing calorie intake (*r* = −0.44; *p* = 0.031), body weight gain (*r* = −0.63; *p* = 0.001) and total visceral adiposity (*r* = −0.77; *p* < 0.001).

Apart from the dilution of the calorie content, cecal fermentation of RS and certain resultant bacterial metabolites (SCFAs) have shown proven effects on improving satiety via several mechanisms [1]. Short chain fatty acids are known to act as signaling molecules in diverse carbohydrate and lipid metabolic pathways, modifying the activites of certain hormones via activation of G-protein coupled receptors (GPRs) that hinder feed intake [1,33]. As revealed by the correlation analysis, RS ingestion was the reason for the higher acetate (*r* = 0.64; *p* = 0.001) and *n*-butyrate (*r* = 0.52; *p* = 0.009) contents observed particularly in the HAS and S-Rf groups. On the other hand, total dietary fiber intake was not significantly correlated with the SCFA content in the cecum, which is understandable due to the neglegible fermentation nature of cellulose, which was the major fraction of total dietary fiber in all diets. However, in this study feed intake was not correlated with either individual or total SCFA contents. Further, body weight gain was not associated with SCFA content, but only with the calorie intake and the intake of RS, as previously mentioned. Thus, it was apparent that RS intake might have potentially diluted calorie intake and subsequently resulted in a lower positive energy balance in the body, as manifested by the lower visceral adiposity and body weight gain.

On the other hand, cecal breakdown of RS and resultant SCFA might have had a significant effect on the reduced visceral fat accumulation in this study, as suggested by the negative correlation between total SCFA content and visceral adipose tissue mass (Figure 5), similar to previous studies [34]. Similarly, acetate and propionate contents were also significantly negatively correlated with the component and total visceral fat masses (Table S3). Profound anti-obesity and anti-diabetic effects of SCFA had been previously reported, as it is found to impart effects on body weight control and fat accumulation via regulation of energy intake and energy expenditure [2,35]. Acetate is reported to regulate appetite via increasing hypothalamic acetyl-CoA carboxylase activity to reduce feed intake and secretion of gut derived satiety hormones through the activation of GPR 41 and 43 [35]. Additionally, propionate administration to obese subjects was also found to increase peptide YY (PYY) and glucagon like peptide (GLP)-1 secretion with significantly reduced adiposity and reduced overall weight gain [1]. *n-*Butyrate was also found to suppress weight gain and reverse/prevent insulin resistance [34]. Further, acetate is also known to affect adipose tissue morphology by influencing the proliferation and differentiation of adipocytes (inhibiting hypertrophy) via the activation of GPR 43, where it mediates anti-lipolytic effects and affect fat storage [2,35,36]. Thus, the negative correlations observed between SCFA and visceral fat mass (Figure 5) might indicate the effects mediated by the above mechanisms. Thus, RS intake had a key role in modulating visceral fat accumulation not only by diluting calorie intake, but also via the effects of colonic breakdown products of RS by gut microbiota in HAS and sorghum fed rats in this study.

In a previous study by the authors, raw sorghum fed rats exhibited contrasting results to the current study, where body weight, visceral fat mass and cecal SCFA profile were similar to the CON group, while the major microbial members were found to be involved in resistant amino acid metabolism instead of RS [15]. In contrast, in this study genus *Ruminococcus* was found to be a key player in the sorghum fed rat cecal microbiota, well known to be able to utilize RS and dietary fiber [37]. The shift in the microbial composition between raw and frozen autoclaved sorghum fed rats could be attributed to the alteration of native physicochemical properties upon autoclaving and subsequent freezing, where RS in sorghum is reported to become more resistant to ileal digestion upon heat treatments and also due to retrogradation during freezing [17]. Further, among various heat treatment methods, autoclaved starches are reported to be more fermentable by amylolytic gut bacteria than raw starches, which could be the reason for the higher *Ruminococcus* abundance in the sorghum fed groups in this study [37].

Short chain fatty acid contents in the sorghum fed groups were only comparatively higher than CON, which could be due to the rapid uptake by the host. Also ten-fold higher RS content in the HAS group could be the reason for the abundantly available SCFA in the cecum at the time of euthanization compared to sorghum fed groups. Further, the higher abundance of *Bifidobacterium* in the HAS group can also be attributed to its higher RS content, as *Bifidobacterium* is known to effectively degrade RS, particularly in high amylose starches [37,38]. Further, the genetic factors that determine the native physicochemical properties (i.e., amylose: amylopectin ratio) of flour/starch and the processing history that alters the native physicochemical properties might have caused different susceptibilities of RS in HAS and sorghum to be degradaded by gut bacteria, which might have been responsible for the observed drastic differences in the major bacterial taxa involved and resultant metabolite contents observed between HAS and sorghum groups [39,40].

The composition of gut microbiota is reported to be a key player in the obese or lean phenotypes [36,41]. Further, reduced bacterial diversity is found to be associated with obesity and overall adiposity, due to altered expression of functional gene content [42]. On the other hand, dietary interventions with prebiotics have shown to modify the composition and functional gene expression of gut microbiota and ameliorate certain metabolic disorders, such as obesity [34]. Higher gut bacterial diversity in sorghum groups might indicate the availability of versatile substrates to cater a wide range of organisms or abundance of functionally redundant organisms and/or cross-feeding organisms. The improved availability and/or accessiblity of RS by heat treatment coupled with freezing could be the reason behind the higher abundance of *Ruminococcus* spp. compared to the previous study that used raw sorghum [15] and lower adiposity might implicate their beneficial physiological effects mediated through the production of SCFA. Further, *Bifidobacterium* and *Akkermansia* are also identified to negatively correlate with the incidence in obesity [36,43]. 

Apart from the adipose tissue, the other major organ involved in lipid metabolism in the body is the liver, where it uptakes chylomicron remnants (dietary fat) and free fatty acids (lipolysis in the adipose tissue), which then are converted into triglycerides and incorporated into lipoproteins [44]. In this study, albeit that the hepatic cholesterol content was significantly higher in S-Rf, serum non-HDL cholesterol content was significantly lower compared to the CON group, and neither were they significantly correlated. Generally, hepatic cholesterol content is a key player in blood LDL-cholesterol content, as it is known to determine the uptake of LDL-cholesterol particles from blood, via determining the number of hepatic LDL receptors [45]. Further, prebiotics are found to inhibit cholesterol synthesis in liver and redistribution from plasma to liver [46,47,48,49]; however, the liver lipid profile and the SCFA profile were not significantly correlated in this study, and instead RS intake was negatively correlated with the liver lipid profile, which could be associated with the calorie diluting effect of RS (Table S3).

Prebiotics have shown promising evidence in improving blood lipid profiles in relation to SCFA production via fermentation of prebiotics and enhancing excretion of cholestrol via feces by increasing viscosity of luminal content [38,48,50,51]. Significantly lower non-HDL cholesterol content in sorghum and HAS fed groups is suggested to be due to the colonic fermentation function of RS intake as backed up by the negative correlation with RS content (*r* = −0.76; *p* < 0.001) and total SCFA content (Figure 5). On the other hand, non-HDL cholesterol was not correlated with the feed, calorie intake or total dietary fiber intake, in contrast to a previous study in humans, which reported otherwise [38]. Thus, the negative correlations observed for non-HDL cholesterol with SCFA content strengthened the cholesterol lowering effect of RS fermentation in sorghum and HAS fed groups. Moreover, lower serum total cholesterol (*r* = −0.71; *p* < 0.001), serum triglyceride (*r* = −0.62; *p* = 0.001) and serum free fatty acid (*r* = −0.66; *p* = 0.001) contents were also significantly correlated with RS intake, not with the colonic fermentation effect, but with the calorie dilution effect (Table S3). As prebiotics influence the growth of probiotics, hypocholesterolemic effects mediated by probiotics also stream parellelly with prebiotic-specific effects, thus cummulatively phenotyped in in vitro and in vivo studies [36]. For example, *Bifidobacterium* and some strains of *Lactobacillus* have shown to redcuce circulating cholesterol, triglyceride and LDL [36].

Gut bacteria are known to mediate lipid homeostasis via various mechanisms, where deconjugation of bile acids and improving excretion via feces, feed-forward conversion of hepatic cholesterol into bile salts by direct binding of cholesterol to cellular membranes, incorporation of cholesterol into cellular membranes during growth and converting into coprostanol and excreting through feces contribute greatly to lowered cholesterol level in blood [47,48,49]. Generally, the primary bile acid synthesis is considered to be determined by the dietary fat intake, yet fat intake and fecal primary bile acid content were negatively correlated (*r* = −0.49; *p* < 0.05) in this study [52]. This could be a result of disparities between the real situation in the duodenum and what is implicated by feces. Furthermore, negative correlation between chenodeoxycholic acid excretion per day and serum total cholestrol (*r* = −0.46; *p* = 0.023), phospholipid (*r* = −0.54; *p* = 0.005), free fatty acid (*r* = −0.48; *p* = 0.019) and non-HDL (*r* = −0.52; *p* = 0.008) could be due to the effects of primary bile acids on cholestrol metabolism [53].

Higher secondary bile acid content observed in the two sorghum fed groups might suggest that a higher proportion of primary bile acids might have been bound to fiber at the duodenum, and transported to the colon without being re-absorbed at the ileum [47]. These primary bile acids might have later been liberated upon fiber fermentation by gut bacteria in the colon and become deconjugated by bacterial bile-salt hydrolases, dehydroxylated and transformed into secondary bile acids by the activity of microbial 7 α-dehydroxylase [47,48,54]. Members of *Clostridium* cluster XVIa have been identified as potential secondary bile acid producers, for example, *Blautia* and *Ruminococcus* spp., thus the higher secondary bile acid content in sorghum groups can be attributed to the higher abundance of *Ruminococcus* spp. [55]. The significant positive correlation observed for the dietary fiber fraction from sorghum with deoxycholic acid (*r* = 0.71; *p* = 0.0001) and total secondary bile acid (*r* = 0.56; *p* = 0.005) contents further backed up the above fact. On the other hand, RS intake was negatively associated with deoxycholic acid (*r* = −0.46; *p* = 0.025) and total secondary bile acid (*r* = 0.50; *p* = 0.014) contents, suggesting the inhibitory effect of RS on secondary bile acid synthesis, which could be the reason behind the negative correlations observed between secondary bile acids and SCFA (Table S3) in this study [56]. Anyhow, the effect of RS might have not been large enough to outcompete the primary bile acid binding effect exerted by the sorghum dietary fiber as implicated by the magnitude of the correlations.

## 5. Conclusions

Sorghum fed rats exhibited a significantly lower visceral adiposity, serum non-HDL cholesterol level, comparatively lower body weight gain and smaller adipocyte size, which were found to be associated with lower calorie intake. Albeit that a clear reason could not be identified for the reduced appetite, the RS content ingested via diet was found to influence a reduced calorie intake, as well as a higher cecal SCFA content. Lower body weight was only associated with reduced calorie intake, while lower visceral fat accumulation was associated with both lower calorie intake and SCFA contents. Further, lower visceral fat accumulation was significantly associated with lower body weight gain. Lower serum non-HDL content was also associated with RS intake, but only associated with cecal SCFA contents, and not with lower calorie intake, suggesting a key role played by the colonic fermentation of RS and its resultant metabolites in lipid metabolism. Albeit that GPR expressions were not determined in this study, the correlations observed for SCFA with adipose mass and serum non-HDL cholesterol implicated a clear involvement of SCFAs in the lipid metabolism in adipose tissue and serum. Thus, autoclaving and freezing of sorghum might have passed down more starch into the colon, influencing the proliferation of beneficial bacterial growth, such as *Ruminococcus* spp. enhancing the colonic breakdown of RS and production of SCFA, which might have exerted beneficial effects, such as lower visceral adiposity and lower non-HDL cholesterol level in serum, ultimately phenotyping a healthy physiological status in sorghum fed rats compared to the negative control group.

## Figures and Tables

**Table 1:** Zoometric parameters, feed intake and organ parameters of rats.

Parameter	CON	HAS	S-Wh	S-Rf
Final body weight (g)	250 ± 5 ^a^	228 ± 5 ^b^	241 ± 5 ^ab^	241 ± 3 ^ab^
Body weight gain (g/30 days)	62.0 ± 3.9 ^a^	40.8 ± 4.4 ^b^	54.0 ± 2.7 ^ab^	53.8 ± 2.8 ^ab^
Feed intake (g/30 days)	384 ± 6 ^a^	357 ± 6 ^b^	349 ± 7 ^b^	341 ± 7 ^b^
Ep-AT (g/100 g body weight)	2.57 ± 0.14 ^a^	1.71 ± 0.06 ^c^	2.17 ± 0.10 ^b^	2.12 ± 0.05 ^b^
Pe-AT (g/100 g body weight)	2.29 ± 0.07 ^a^	1.46 ± 0.09 ^c^	1.99 ± 0.12 ^b^	1.94 ± 0.10 ^b^
Tv-AT (g/100 g body weight)	4.86 ± 0.20 ^a^	3.17 ± 0.13 ^c^	4.17 ± 0.22 ^b^	4.06 ± 0.12 ^b^
Cecal weight (g)	2.32 ± 0.22 ^b^	5.80 ± 1.37 ^a^	1.99 ± 0.08 ^b^	2.14 ± 0.13 ^b^
Cecal tissue weight (g)	0.67 ± 0.02 ^b^	1.27 ± 0.09 ^a^	0.67± 0.05 ^b^	0.65 ± 0.02 ^b^
Cecal content weight (g)	1.65 ± 0.21 ^b^	4.52 ± 1.28 ^a^	1.31 ± 0.10 ^b^	1.48 ± 0.12 ^b^

Values presented are mean ± SE (*n* = 6); values followed by different lowercase letters are significantly (*p* < 0.05) different. (Abbreviations: CON, α-corn starch; HAS, high amylose starch; S-Wh, frozen cooked whole white sorghum; S-Rf, frozen cooked refined white sorghum; Ep-AT, epididymal adipose tissue; Pe-AT, perirenal adipose tissue; Tv-AT, total visceral adipose tissue).

**Table 2:** Cecal biochemical parameters.

Parameter	CON	HAS	S-Wh	S-Rf
Acetate (µmol/cecum)	118 ± 24 ^b^	271 ± 58 ^a^	98 ± 12 ^b^	152 ± 27 ^ab^
Propionate (µmol/cecum)	14.7 ± 2.3 ^ns^	29.2 ± 9.7 ^ns^	13.9 ± 2.0 ^ns^	19.0 ± 3.3 ^ns^
*n*-Butyrate (µmol/cecum)	2.76 ± 0.70 ^ab^	5.50 ± 0.67 ^a^	0.94 ± 0.19 ^b^	3.85 ± 1.25 ^ab^
Total SCFA (µmol/cecum)	136 ± 27 ^ab^	306 ± 67 ^a^	113 ± 14 ^b^	175 ± 31 ^ab^
pH	7.72 ± 0.10 ^a^	7.30 ± 0.11 ^b^	7.71 ± 0.06 ^a^	7.67 ± 0.11 ^ab^

Values presented are mean ± SE (*n* = 6); values followed by different lowercase letters are significantly (*p* < 0.05) different. (Abbreviations: CON, α-corn starch; HAS, high amylose starch; S-Wh, frozen cooked whole white sorghum; S-Rf, frozen cooked refined white sorghum; ns, not significant).

**Table 3:** Liver lipid, fecal dry matter, moisture, lipid and bile acid contents.

Parameter	CON	HAS	S-Wh	S-Rf
Liver lipid parameters				
Liver weight (g)	5.91 ± 0.07 ^ns^	5.85 ± 0.22 ^ns^	5.80 ± 0.13 ^ns^	5.83 ± 0.08 ^ns^
Liver total lipids (mg)	305 ± 32 ^ns^	324 ± 26 ^ns^	430 ± 49 ^ns^	305 ± 40 ^ns^
Liver cholesterol (mg)	21.67 ± 1.32 ^ab^	19.32 ± 0.68 ^b^	20.91 ± 0.90 ^ab^	23.90 ± 0.88 ^a^
Liver triglyceride (mg)	56.18 ± 3.92 ^ns^	46.86 ± 3.26 ^ns^	56.23 ± 3.30 ^ns^	55.34 ± 3.42 ^ns^
Fecal parameters				
Fecal dry weight (g)	0.91 ± 0.03 ^ab^	1.16 ± 0.18 ^a^	0.77 ± 0.02 ^b^	0.76 ± 0.03 ^b^
Fecal moisture content (%)	54.7 ± 3.5 ^ns^	63.3 ± 4.6 ^ns^	59.6 ± 2.9 ^ns^	51.9 ± 2.3 ^ns^
Fecal total lipids (mg/day)	29.8 ± 4.8 ^ns^	58.6 ± 12.0 ^ns^	38.1 ± 8.8 ^ns^	27.7 ± 7.1 ^ns^
Fecal neutral sterol (mg/day)	2.09 ± 0.26 ^ns^	2.30 ± 0.42 ^ns^	1.69 ± 0.23 ^ns^	2.27 ± 0.23 ^ns^
Fecal cholesterol (mg/day)	0.90 ± 0.10 ^ns^	0.60 ± 0.13 ^ns^	0.75 ± 0.06 ^ns^	1.08 ± 0.10 ^ns^
Fecal coprostanol (mg/day)	0.98 ± 0.25 ^ns^	1.39 ± 0.43 ^ns^	0.77 ± 0.17 ^ns^	1.00 ± 0.08 ^ns^
Fecal bile acid content (mg/day)				
Cholic acid	0.38 ± 0.07 ^ns^	0.93 ± 0.23 ^ns^	0.85 ± 0.20 ^ns^	0.57 ± 0.13 ^ns^
Chenodeoxycholic acid	<0.01 ^ns^	0.07 ± 0.04 ^ns^	0.01 ± 0.00 ^ns^	<0.01 ^ns^
Primary bile acid content	0.46 ± 0.11 ^ns^	1.19 ± 0.30 ^ns^	1.04 ± 0.14 ^ns^	0.69 ± 0.19 ^ns^
Deoxycholic acid	0.46 ± 0.06 ^b^	0.34 ± 0.07 ^b^	0.79 ± 0.09 ^a^	0.56 ± 0.06 ^ab^
Lithocholic acid	0.61 ± 0.13 ^b^	0.41 ± 0.10 ^b^	0.92 ± 0.11 ^ab^	1.21 ± 0.34 ^a^
Secondary bile acid content	1.15 ± 0.21 ^ab^	0.81 ± 0.15 ^b^	1.85 ± 0.20 ^a^	1.88 ± 0.29 ^a^
Total bile acid content	1.61 ± 0.29 ^b^	2.01 ± 0.40 ^ab^	2.89 ± 0.32 ^a^	2.57 ± 0.31 ^a^

Values presented are mean ± SE (*n* = 6); values followed by different lowercase letters are significantly (*p* < 0.05) different. (Abbreviations: CON, α-corn starch; HAS, high amylose starch; S-Wh, frozen cooked whole white sorghum; S-Rf, frozen cooked refined white sorghum; ns, not significant).

## Supplementary Materials

The following are available online at https://www.mdpi.com/2072-6643/12/8/2412/s1, Table S1: Proximate composition of the frozen autoclaved sorghum. Table S2: Composition of experimental diets, Table S3: Pearson’s correlation coefficients.
